# Does the availability of influenza vaccine at prenatal care visits and of immediate vaccination improve vaccination coverage of pregnant women?

**DOI:** 10.1371/journal.pone.0220705

**Published:** 2019-08-01

**Authors:** Vivien Alessandrini, Olivia Anselem, Aude Girault, Laurent Mandelbrot, Dominique Luton, Odile Launay, François Goffinet

**Affiliations:** 1 Maternité Port-Royal, Université Paris Descartes, Groupe hospitalier Cochin Broca Hôtel-Dieu, Assistance Publique-Hôpitaux de Paris, Paris, France; 2 DHU Risques et Grossesse, PRES Sorbonne Paris Cité, Paris, France; 3 Maternité Louis-Mourier, Université Diderot Paris 7, Hôpitaux Universitaires Paris Nord Val-de-Seine, Assistance Publique Hôpitaux de Paris, Colombes, France; 4 Maternité Bichat-Claude Bernard, Université Diderot Paris 7, Hôpitaux Universitaires Paris Nord Val-de-Seine, Assistance Publique Hôpitaux de Paris, Paris, France; 5 Université Paris Descartes, Sorbonne Paris Cité, Inserm, CIC 1417, AP-HP, Groupe Hospitalier Cochin-Broca-Hôtel-Dieu, Centre d’investigation Clinique Cochin-Pasteur,Paris, France; 6 Unité INSERM U953, Recherche épidémiologique en santé périnatale et santé des femmes et des enfants, UPMC, Paris, France; University of Campania, ITALY

## Abstract

**Introduction:**

Although vaccination against influenza is recommended for pregnant women in France because it exposes them to a risk of death and severe respiratory complications, their vaccination coverage in 2016 was estimated at 7%. This study's principal objective was to assess the association between the availability of influenza vaccination at prenatal care visits and vaccination coverage.

**Material and methods:**

This multicenter survey took place in 3 Paris-area public hospital (AP-HP) maternity wards (A, B, and C). Only maternity ward A offered the vaccine and vaccination without charge at prenatal visits. Data were collected from parturients during 10 days in January 2017 by a self-administered anonymous questionnaire.

**Results:**

Data from 248 women showed overall vaccination coverage of 19.4% (48/248): 35.4% (46/130) in maternity unit A, 2.7% (2/75) in B, and 0% (0/43) in C (P<0.01). After adjustment for socio-demographic characteristics, women at maternity ward A were significantly more likely to be vaccinated than those at B and C (aOR 25.52, 95%CI [5.76–113.10]). Other factors significantly associated with higher vaccination coverage were the mother’s French birth (aOR 2.37 CI [1.03–5.46]) and previous influenza vaccination (aOR 3.13, 95%CI [1.25–7.86]). Vaccinated women generally considered they had received adequate information (aOR 4.15 CI [2.10–8.22]), principally from the professional providing their prenatal care. Nonvaccination was attributed to the absence of an offer of vaccination (81.5%), fear of fetal side effects (59.5%), and inadequate information (51.4%).

**Conclusion:**

Our results show that availability of influenza vaccination, free of charge, at prenatal consultations at the maternity ward increases vaccination coverage significantly.

## Introduction

Pregnant women who develop influenza are at higher risk of death and severe respiratory complications than nonpregnant women of the same age [1–6]. During the 2009 pandemic, pregnant women with the H1N1 virus were four times as likely as nonpregnant women to be hospitalized [1]; they also accounted for 4 to 13% of the deaths associated with pandemic influenza infection [1–5]. Similarly, during the 2010–2011 season in France, 35 pregnant women with no comorbidities were admitted to intensive care units; they represented 4% of all severe cases of seasonal influenza [6]. Besides the maternal risk, the onset of influenza during pregnancy, like that of other systemic infections, leads to higher risks of spontaneous abortion, in utero fetal death, and threatened preterm delivery [7–9].

Influenza vaccination is effective in pregnant women [10–13] and has been shown to be safe for both mothers and newborns in several large cohorts and national registries [14–16]. Several studies have also suggested that influenza vaccination of pregnant mothers is associated with a lower risk of preterm birth, small-for-gestational-age status, and stillbirth [17–18].

Influenza vaccination during pregnancy is also beneficial to the fetus, because the well-documented transplacental passage of maternal Ig G antibodies provides protection for newborns and infants who cannot be vaccinated before the age of 6 months [10–13, 19–21].

Vaccination against influenza has thus been recommended since 2009 in France for pregnant women, regardless of the term of their pregnancy at the time of the vaccination campaign. Nonetheless, influenza vaccination coverage in pregnant women in France is very insufficient: 29.3% during the 2009 A/H1N1 pandemic and 7% in 2016 [22–23].

According to the literature, factors associated with influenza vaccination appear to be its offer by healthcare providers [24–32] but also, as our previous work has shown, sociodemographic characteristics and previous vaccination against this virus, especially for patients with one or more comorbidities [30,33]. Among the factors that may explain low vaccination coverage, vaccine hesitation involves many variables that influence the final decision to accept, delay, or refuse vaccines [34–38]. This issue is a problem in many countries but especially in France, which has one of the highest prevalence rates of vaccine hesitancy in the world [39]. Similarly, difficulty of access to the vaccine is a factor that can limit adherence to it. Several studies have accordingly tested different models of offering the vaccine, and some have significantly improved vaccination coverage [40–42].

Thus, one perinatal center decided to make seasonal influenza vaccination available and free of charge during prenatal visits and to assess the impact of this measure on vaccination coverage among all of the women receiving care there.

The principal objective of this study was to assess the association between the availability of influenza vaccine at prenatal visits and vaccination coverage.

## Material and methods

This cross-sectional, multicenter survey took place at three maternity units (Port-Royal Hospital, unit A, Louis-Mourier Hospital, unit B, and Bichat Claude Bernard Hospital, unit C), all belonging to the AP-HP (Assistance Publique-Hopitaux de Paris), the Paris public (and teaching) hospital system, one of the largest in France. These three maternity units were selected because they share common practices and protocols as part of the Pregnancy and Risk center of excellence, which federates various clinical, research, and teaching units from several hospitals, universities, and laboratories on this topic, for which its expertise is widely recognized. These three maternity units were located in distinctive regions of Ile-de-France, each with its own social and demographic specificities.

All three maternity units applied the same guidelines concerning influenza vaccination during the 2016–2017 vaccination campaign. Maternity unit A nonetheless undertook a supplementary action, modifying its organization of care to improve vaccination coverage: it made vaccines available at consultations, free of charge. More precisely, since 2015, the women receiving prenatal care there have been able to be vaccinated by a nurse after a prenatal care visit, if their midwife or physician has put a prescription in the medical file. In the other two hospitals (B and C), free vaccination was not available during consultations; instead, the midwife or physician could write a prescription, to be purchased afterwards by the patient at a pharmacy, and then administered by a general practitioner, other physician, midwife, or nurse in private practice, or by the consultant during the next visit. Although the purchase of the vaccine at a pharmacy is free, its injection by a healthcare provider in private practice is reimbursed at 100% only from the first day of the sixth month of pregnancy and thereafter. Before this point in pregnancy, the reimbursement rate ranges from 60 to 70%, depending on the type of provider consulted.

The objective was therefore to test the effect of making the vaccine available, free of charge, at prenatal consultations on the vaccination coverage of women giving birth at the maternity ward.

Anonymous self-administered questionnaires were distributed before discharge to all women who gave birth during a 10-day period (January 9 to 18, 2017) [S1 Appendix]. This period was chosen so that all the women included would have had prenatal consultations during the 2016–2017 influenza vaccination campaign, which took place in France from October 7, 2016, to January 31, 2017. The influenza epidemic began in mid-December and ended in early February [43]. The questionnaires were developed by gynecologists-obstetricians and infectious disease specialists. Some of the items covered vaccination determinants previously validated in other studies (vaccination offer, previous vaccination, sociodemographic characteristics [24–33]), while others attempted to identify determinants that have been studied less (source and quality of the information received, general perception of vaccines, perception of the seriousness of influenza for mother and baby, and perception of the vaccine’s safety during pregnancy).

The questionnaire was then pilot-tested a month before the start of the study, to ensure its clarity and comprehensibility. The investigator who distributed the main questionnaire also distributed the pilot test to 10 parturients; it was subsequently modified to improve the issues noted.

The National Data Protection Authority (CNIL n° 1755849) approved this study. They were also informed that their records could be used for the evaluation of medical practices and were offered the choice of opting out of such studies.

The final questionnaires were distributed, explained if necessary, and collected by the same person, at each of the three maternity units. The women completed the questionnaires themselves. All women recruited were included regardless of their term at delivery, except that women who did not speak French were excluded.

We obtained the number of women giving birth at each hospital during the study period to assess the questionnaire response rate. Then we collected the following information from the questionnaire: women's vaccination status, age, geographic origin, number of children living at home, singleton or multiple pregnancy, smoking status, any other chronic disease that is itself an indication for influenza vaccination (chronic cardiac, pulmonary, hepatic, renal or hematologic disease, hemoglobin disorder, preexisting diabetes, cancer, and immune deficiencies including HIV), previous influenza vaccination, social-occupational category, and whether their occupation involved health care or contact with children or the public. The occupational categories were classified according to the INSEE (National institute of statistics and economic studies) classification [S2 Appendix] and then reorganized into 3 groups: one group combining company heads, managers and professionals; a second group bringing together intermediate white-collar occupations, crafts workers and tradespeople, shopkeepers, and office, sales, and service workers; and a third group of farmers, manual workers, the unemployed, and people not in the labor force.

To study other determinants of influenza vaccination, we used questionnaire responses to assess whether women considered that they had received adequate information about this vaccination and what sources of information they had used: medical professionals during prenatal or other medical consultations, internet and media, family and friends, or none. Their opinions about the effectiveness of vaccines generally and the frequency of adverse side effects were also recorded. To examine whether perceptions of the seriousness of influenza affected adherence to the vaccine, women were asked whether influenza is a serious disease for several different categories of people: pregnant women, nonpregnant women, women with a chronic disease, and newborns. Potential reasons for not being vaccinated were listed next, with respondents asked to select as many of the following reasons as they considered applicable: no one offered them vaccination, fear of side effects for self or fetus, doubt about this vaccine's effectiveness, general antivaccine beliefs, difficult access to the vaccine, or other (free responses). These items were also studied for women who were offered but refused vaccination. Finally, we asked which consultant had proposed the vaccine (physician at the maternity ward or in private practice, midwife, other).

The analysis began by a comparison of the sociodemographic characteristics of the populations of each of the three maternity units. Qualitative variables were compared by the chi-square test or Fisher's exact test, as appropriate. The quantitative variables were compared with Student's t test. To assess the determinants of vaccination, sociodemographic characteristics and perceptions of vaccines in general were compared between the women who were and were not vaccinated. We decided to combine hospitals B and C for the multivariate analyses because the vaccination policy in these two maternity units was similar, with in particular the influenza vaccine unavailable for administration at the prenatal consultation. We ran two multivariate logistic regression models, one of which included the sociodemographic variables from the questionnaire, and the other with the variables associated with vaccine perceptions. All factors with a P value <0.20 in the univariate analysis were included in the multivariate analysis. We chose to make two different logistic regression models, to avoid a potential risk of overadjustment. Nonetheless, we also performed a global logistic regression including all of the social and demographic factors and those associated with vaccine perceptions [S1 Table]. These models enabled us to obtain adjusted ORs (aOR) and their 95% CIs.

Statistical significance was defined as P < 0.05. The statistical analyses were performed with Stata software, version 13.0.

## Results

### Centers' vaccination rates and social and demographic characteristics

During the study period, 248 women completed the questionnaire: 130 women in hospital A, 75 in hospital B, and 43 in hospital C, for an overall mean response rate of 66.0%: 63.4% (A), 80.6% (B), and 55.1% (C). The mean vaccination coverage for all 3 units was 19.4% (48/248): 35.4% (46/130, including 38 vaccinated at the maternity unit) (A), 2.7% (2/75) (B), and 0% (0/43) (C) [Table 1].

**Table 1:** Comparison of the sociodemographic characteristics of the women giving birth in maternity units A, B, and C (N = 248).

	Maternity A N = 130 n (%)	Maternity B N = 75 n (%)	Maternity C N = 43 n (%)	*P*
**Women hospitalized during the study period**	205	93	78	
**Response rate**	63.4	80.6	55.1	
**Vaccination against influenza**	46 (35.4)	2 (2.7)	0	<0.01
**Age (years), (mean ± SD)**	33.1 ± 5.1	32.6 ± 5.5	32.7 ± 5.3	0.78
**Geographic origin**				
France	68 (52.3)	33 (44.0)	13 (30.2)	
Sub-Saharan Africa	26 (20.0)	13 (17.3)	15 (34.9)	
West Indies	1 (0.7)	3 (4.0)	0 (0.0)	<0.01
North Africa	26 (20.0)	24 (32.0)	9 (20.9)	
Asia	5 (3.8)	1 (1.3)	0 (0.0)	
Other	4 (3.1)	1 (1.3)	6 (14.0)	
**Number of children (mean ± SD)**	0.8 ± 1.1	1.4 ± 1.3	1.3 ± 1.1	<0.01
**Twin pregnancy**	5 (3.8)	4 (5.3)	0	0.39
**Smoked**				
During pregnancy	7 (5.4)	5 (6.7)	1 (2.3)	
Before pregnancy	10 (7.7)	8 (10.7)	2 (4.7)	0.63
Nonsmoker	113 (86.9)	62 (82.7)	40 (93.0)	
Comorbidities*	7 (5.4)	6 (8)	2 (4.7)	0.77
Previous influenza vaccination**	35 (27.1)	12 (16)	5 (11.9)	0.06
**Social-occupational category**				
Company heads, managers, professionals	51 (39.2)	8 (10.7)	7 (16.3)	
Intermediate white-collar occupations, shopkeepers, crafts workers, tradespeople, office, sales, and service workers	50 (38.5)	44 (58.7)	13 (30.2)	<0.01
Farmers, manual workers, the unemployed, and people not in the labor force	29 (22.3)	23 (30.7)	23 (53.5)	
**Healthcare worker**	18 (13.8)	4 (5.3)	4 (9.3)	0.16
**Occupation working with children**	33 (25.4)	16 (21.3)	4 (9.3)	0.08
**Occupation in contact with the public**	42 (32.3)	26 (34.7)	6 (14.0)	0.03

Mean ± SD: mean ± standard deviation. The percentages are indicated between parentheses.

* chronic cardiac, pulmonary, hepatic, renal or hematologic disease, hemoglobin disorder, diabetes, cancer, and immune deficiencies, including HIV.

** Two women did not answer this question.

Table 1 summarizes the sociodemographic characteristics of the women who completed the questionnaire. More than half the women in hospital A (52.3%) were born in metropolitan France, whereas majorities of those in hospitals B (56%) and C (69.8%) came from other countries (P <0.01). Women giving birth at hospital A also had the fewest children at home (0.8±1.1, P <0.05), as well as the highest proportion of women in the highest socio-occupational category (39.2%, P <0.01). Around one third of the women at hospitals A (32.3%) and B (34.7%) (P <0.05) worked in occupations in contact with the public.

### Social and demographic determinants

After adjustment for sociodemographic characteristics, the women receiving care at maternity ward A remained significantly more likely to have been vaccinated than those at units B and C (aOR 25.52 95% CI [5.76–113.10]). The other factors significantly associated with higher vaccination coverage were maternal birth in France (aOR 2.37, 95%CI [1.03–5.46]) and previous influenza vaccination (aOR 3.13, 95% [1.25–7.86] [Table 2].

**Table 2:** Determinants associated with vaccination: Multivariate analysis including maternity ward and social and demographic characteristics (N = 248).

Variables	Vaccinated women N = 48 n (%)	aOR	95% CI
**Maternity ward**			
B and C, n = 118	2 (1.7)	Ref.	-
A, n = 130	46 (35.4)	25.52	(5.76–113.10)
**Geographic origin**			
Other, n = 134	14 (10.4)	Ref.	-
Metropolitan France, n = 114	34 (29.8)	2.37	(1.03–5.46)
**Number of children**			
None, n = 97	27 (27.8)	Ref.	-
1, n = 66	10 (15.2)	0.80	(0.31–2.09)
≥ 2, n = 85	11 (13.1)	0.71	(0.27–1.91)
**Twin pregnancy**			
No, n = 239	44 (18.4)	Ref.	-
Yes, n = 9	4 (44.4)	3.39	(0.43–26.69)
Previous influenza vaccination*			
No, n = 195	27 (13.8)	Ref.	-
Yes, n = 51	21 (41.2)	3.13	(1.25–7.86)
**Healthcare workers**			
No, n = 222	38 (17.1)	Ref.	-
Yes, n = 26	10 (38.5)	0.60	(0.18–2.03)
**Social-occupational category**			
Company heads, managers, professionals, n = 66	24 (36.4)	Ref.	-
Intermediate professions, tradespeople, crafts workers and shopkeepers, office, sales, and service workers, n = 107	17 (15.9)	0.80	(0.33–1.95)
Farmers, blue-collar workers, unemployed, not in the labor force n = 75	7 (9.3)	0.71	(0.23–2.18)

aOR: adjusted odds ratio; 95% CI%: 95% confidence interval

*Two women did not answer this question.

### Determinants associated with perception of vaccination

Overall, 22% of the nonvaccinated women felt that they had received adequate information about the vaccine, compared with 54.2% of the vaccinated women (*P*<0.01). After adjustment, the vaccinated women considered still more strongly that they had been adequately informed (aOR 4.15 95% CI [2.10–8.22]) [Table 3]. The principal source of information for the vaccinated women was their prenatal care provider (aOR 9.5 CI [3.30–27.30]).

**Table 3:** Determinants associated with vaccination: Multivariate analysis including the variables associated with perception of vaccination (N = 248).

Variables	Vaccinated women N = 48 n (%)	aOR	95% CI
**Consider they received enough information, n = 70**			
No, n = 178	22 (12.3)	Ref.	-
Yes, n = 70	26 (37.1)	4.15	(2.10–8.22)
**Sources of information**			
None, n = 44	0 (0.0)	-	-
In consultation, n = 77	40 (51.9)	9.5	(3.30–27.30)
Family and friends, n = 66	12 (18.2)	0.72	(0.29–1.78)
Media and internet, n = 118	13 (11.0)	0.60	(0.23–1.54)
**Think that vaccines are**			
Ineffective or not very effective, n = 42	5 (11.9)	Ref.	-
Effective or very effective, n = 206	43 (20.9)	1.40	(0.48–4.14)
**Think that the side effects of vaccines are**			
Frequent or very frequent, n = 93	9 (5.8)	Ref.	-
Very rare or rare, n = 155	39 (41.9)	2.87	(1.25–6.60)

aOR: adjusted odds ratio; 95% CI%: 95% confidence interval.

Ref.: reference

The vaccinated and nonvaccinated groups had similar perceptions of the effectiveness of the vaccine (aOR 1.40, 95% CI [0.48–4.14]), but a significantly higher proportion of nonvaccinated women felt that vaccines are responsible for frequent side effects (aOR 2.87, 95%CI [1.25–6.60]).

The vaccinated and nonvaccinated women did not differ significantly in their opinion of influenza as a serious disease outside pregnancy (45.8% vs 51%, P = 0.31), during pregnancy (91.7% vs 81.5%, P = 0.06), for a person with a chronic disease (91.7% vs 88%, P = 0.33), or for newborns (98% vs 92%, P = 0.12).

### Factors associated with the lack of vaccination

The principal reason reported for nonvaccination was the absence of any offer of vaccination in 81.5% of cases (67.9% in maternity unit A, 91.8% in B, and 90.7% in C, P<0.001). Other factors associated with nonvaccination were: fear of side effects for the fetus (28.5%) and for the mother (17%), doubt about the vaccine's effectiveness (15%), general opposition to vaccines (8.5%), other reasons (2%), and difficulty of access to the vaccine (1%).

When the vaccine was offered, the factors associated with refusal were fear of side effects for the fetus (59.5%), insufficient information (51.4%), fear of personal side effects (43.2%), doubt about its effectiveness (27%), general opposition to vaccines (18.9%), and difficulty of access to the vaccine (2.7%).

The results of the multivariate analysis including both the sociodemographic factors and those related to perceptions of the vaccine are similar [S1 Table].

Finally, the different categories of healthcare providers offered the vaccine at rates that did not differ significantly: hospital staff physicians (41.6%), private practice physicians (8.3%), midwives (43.8%), and others (6.3%) (P = 0.38).

## Discussion

In our study, the availability of the vaccine free of charge at prenatal consultations at the maternity ward appeared to improve vaccination coverage. Other factors associated with higher vaccination coverage were birth in mainland France and previous vaccination against influenza. The obstacles to vaccine uptake were the absence of any offer of the vaccine, lack of adequate information, and the fear of side effects.

One of the strong points of our study is that it is a multicenter survey, including 248 women over the same period. This allowed us to include a diverse population. Moreover, we obtained a satisfactory response rate (66%), because we had a staff person assigned to the distribution and collection of the questionnaires. We note the unequal number of participants from each maternity unit, due mainly to the lower numbers of annual deliveries at maternity units B and C and the lower response rate at maternity unit C (explained in part by the higher number of women who did not speak French). Accordingly, the rates reported may not reflect the actual vaccination rates, in particular at hospital C. Although we do not know the details of the reasons for non-inclusion at each center (did not speak French, refused to complete questionnaire, did not return the questionnaire), it is probable that the characteristics of the women not included at hospitals B and C were those associated with a low vaccination rate (did not speak French and low socioeconomic status). This would have resulted in an underestimation of the difference in the vaccination rates that we observed between center A and centers B and C.

The social and demographic characteristics of the women differed between the three maternity units, as expected. Nonetheless, although geographic origin might influence vaccine adherence, we were not seeking to identify the factors associated with nonvaccination but simply to control for them in order to be able to achieve our objective. Finally, although the characteristics of the women included in our study may be markedly different from those of women in other French hospitals or in other industrialized countries, we do not think that this calls into question our result about the association between the availability of the vaccine and the improvement of vaccination coverage in the center, given that we took most of the confounding factors into account.

Another limitation of our survey was the period chosen for the distribution of the questionnaires; the choice of a period later during the vaccination campaign would have allowed us to include women seen more often during the campaign, who would therefore have had more opportunities to be offered the vaccine. Nonetheless, the study period took place three months after the beginning of the vaccination campaign, so that each woman should have had around 3 visits during which vaccination should have been offered. Moreover, this choice of study period also meant that most of the women vaccinated during our study received their injections at the end of the second or during the third trimester. The extrapolation of our results for first-trimester vaccination is thus questionable.

The principal interest of our survey was the comparison of three maternity units that differed according to their influenza vaccination policy, with one offering immediate and free vaccination, and the other two following the more complicated standard procedure, without any other differences in their organization of prenatal care. The significant difference in vaccination coverage between the three units according to whether the vaccination was immediately available or simply prescribed shows that making immediate vaccination possible leads to substantially higher vaccination coverage. In a Canadian study, making the influenza vaccine available also improved influenza vaccination coverage in a maternity ward [44]. It is nonetheless possible that the intention and desire to vaccinate was stronger among staff at maternity unit A, independent of the availability of the vaccine at prenatal visits. It is unfortunately not possible to distinguish these two effects, and this impossibility limits the interpretation of our results.

Our results show the same determinants of influenza vaccination identified in other studies: a history of influenza vaccination, which improved vaccine use, and geographic origin, with lower vaccination coverage among people born abroad [33,45,46]. Beyond the language barrier and limited access to media, which may explain the deficit in vaccination in this population, the financial aspect must also be considered; reimbursement for the vaccine is a major determinant of access to vaccination [47]. Moreover, although they are fully reimbursed in France, the need to pay these costs in advance can be a barrier, one that can be overcome by making the vaccination immediately available and free of charge, so that the woman does not have to lay out money. Moreover, the availability of the vaccine also facilitates immediate vaccination, so that the women are not required to find or visit another healthcare professional to be vaccinated.

When the vaccine is offered, the identification of factors that may influence vaccine hesitancy or even refusal is essential to be able to design measures to improve it. Accordingly, several studies have shown greater reluctance to accept influenza vaccination among nulliparas, women with a lower educational level, and those not living in their native country [38, 48–49]. According to these data and our results, information about its safety for both mother and fetus is essential, especially among the subgroups mentioned. Several studies have shown improved adherence among pregnant women well informed of the dual benefit of vaccination, for themselves and their fetuses [50–54]. It is also possible that the institutional provision of the vaccine will reassure women about its safety. Its availability at a hospital or other medical consultation underlines the consensus among healthcare professionals about its effectiveness and safety to women who have doubts about it.

Among the unvaccinated pregnant women, the principal reason for nonvaccination remained that no healthcare provider had offered vaccination. The importance of a healthcare professional's recommendation of vaccination in our study confirms previously reported results [24–32]. In one of these studies, 67.3% of the pregnant women questioned reported that vaccination against influenza was recommended and offered by their clinicians, 11.9% that it was recommended but not offered, and 20.7% received neither a recommendation nor an offer of vaccination; their respective vaccination rates were 70.5%, 43.7%, and 14.8% [24].

This points out the need to train staff to promote vaccination acceptance by both women and healthcare workers. A US study of obstetricians' practices in the prescription of the vaccine against whooping cough illustrates this point: 92% knew the guidelines, 80% recommended the vaccination, and finally 67% offered it at visits [55]. One solution to the absence of an offer is to make the vaccine and vaccination immediately available. In particular, making the vaccine available probably plays a role in informing and sensitizing healthcare workers and thus influences their offer. This effect can tend to be reinforced over time with the progressive dissemination of guidelines and increased awareness by medical professionals; they thus tend to prescribe the vaccine increasingly routinely each year.

Beyond individual information to women and the willingness of healthcare professionals, recommendations by the public authorities can have an impact. Accordingly, among the measures aimed at improving vaccination coverage in France that went into effect on January 1, 2018, the French government made eight additional vaccines mandatory. Although the vaccine against influenza is not mandatory [56], this attitude appears to be having a positive impact on vaccination coverage and may in the long term also influence the vaccine adherence of the populations not directly concerned by the change [57].

Similarly, making flyers available to women in maternity units, stressing the benefits and safety of the vaccine, may also improve vaccination coverage, offering a supplementary source of information. The media, a source of information for a great number of patients, might usefully provide information about this vaccine's effectiveness and safety during each vaccination campaign, in addition to providing hygiene advice.

## Conclusion

In our study, the change in the organization of care to make the vaccine and immediate immunization available, free of charge, at maternity unit A enabled a significant increase in vaccination coverage—from 0 to 35.4%. The failure of healthcare providers to recommend and offer the vaccine at the time of prenatal care, lack of information, and fear of side effects were the principal obstacles to vaccination against influenza.

The simplification of the vaccination process is probably one of the reasons for the success of this measure, by limiting the number of missed occasions. But it also is part of an especially active and assiduous vaccination campaign. Generalizing the free availability of vaccine on a larger scale would probably make it possible to observe progress in care providers' practices. That is, by making the prescription of vaccine routine and playing a role in informing prescribers, it should contribute to making vaccination a sustainable practice in maternity units.

## Supporting information

S1 Appendix Self-administered questionnaire. (DOCX)

S2 Appendix Correspondence between the different levels of the classification of occupations and the INSEE occupational category (2003). (DOCX)

S1 Table Sociodemographic determinants and pregnant women's perceptions of influenza and vaccination, multivariate analysis (N = 248). aOR: adjusted odds ratio; 95% CI%: 95% confidence interval. Ref.: reference. Adjusted for all sociodemographic variables and variables associated with perceptions of vaccines with p value <0.20 in univariate analysis.
(DOCX)
