# Supplementary material for: Does the availability of influenza vaccine at prenatal care visits and of immediate vaccination improve vaccination coverage of pregnant women?
Source: PLoS One. 2019 Aug 1;14(8):e0220705. doi: 10.1371/journal.pone.0220705 (PMC6675112; doi:10.1371/journal.pone.0220705)
Supplement: S1 Appendix — (DOCX) [file pone.0220705.s001.docx]

**Appendix S1.**

**Self-administered questionnaire**

**INFLUENZA AND PREGNANCY**

**Were you vaccinated against influenza during the winter of 2016/2017?**

□ Yes No

**Month of pregnancy at vaccination**: ………………………………………

**If YES, where were you vaccinated?**

- at the maternity ward
- at your general practitioner's office
- at the occupational physician's office
- elsewhere: ……………………….

**If you were vaccinated at the maternity ward, do you think that you would have been vaccinated if the vaccine had not been available at your appointment and you had had to go elsewhere?**

□ Yes □ No □ don't know

**If not, for what reason(s)?**

- not offered
- doubt about its efficacy
- fear of side effects for you
- fear of side effects for the baby
- access to the vaccine appeared difficult
- generally anti-vaccine
- other:………………….

**Who offered you the vaccination?**

- a physician at the maternity ward
- A physician in private practice
- a midwife
- other: …………

**Do you think that the flu can be a serious disease?**

- for women who are not pregnant □ Yes □ No
- for pregnant women □ Yes □ No
- for a woman, pregnant or not, with a chronic disease □ Yes □ No
- for a newborn □ Yes □ No

**Do you feel you received enough information about this vaccination?**

□ Yes No

**What were your sources of information?**

- Internet and media
- At prenatal visits
- family and friends
- other: …………

**Other than during this pregnancy, have you ever been vaccinated against the flu?**

□ Yes No

**Generally, do you think that vaccines are effective in preventing infections?**

□ very effective □ effective □ not very effective □ ineffective

**Do you think that vaccines cause side effects?**

□ very frequently □ frequently □ rarely □ very rarely

**Have you received information about vaccination against whooping cough, for you and your family?** □ Yes □ No

**Would you have been in favor of a vaccination during your pregnancy to protect your newborn against:**

- whooping cough □ Yes □ No □ Don't know
- the virus that causes bronchiolitis □ Yes □ No □ Don't know

**Age:**

………………………………………………………………………………………...……………………………

**Medical history:** ………………………………………………………………...………………………………..…

**Number of children** **at home:** ……………………………………………………………………...……………………………

**Occupation:** ……………………………………………………………………………...………………………………

**Geographic origin**:

🞎 Metropolitan France 🞎 North Africa

🞎 Sub-Saharan Africa 🞎Asia

🞎 West Indies 🞎 other:……………………………

**Current pregnancy:**

🞎 singleton 🞎 twins or triplets

**Did you smoke before this pregnancy?** 🞎 Yes 🞎 No If yes, number of cigarettes a day: ……….

**Did you smoke during this pregnancy?** 🞎 Yes 🞎 No If yes, number of cigarettes a day: ……….
