# Supplementary material for: Does the availability of influenza vaccine at prenatal care visits and of immediate vaccination improve vaccination coverage of pregnant women?
Source: PLoS One. 2019 Aug 1;14(8):e0220705. doi: 10.1371/journal.pone.0220705 (PMC6675112; doi:10.1371/journal.pone.0220705)
Supplement: S2 Appendix — (DOCX) [file pone.0220705.s002.docx]

**Appendix S2.**

**Correspondence between the different levels of the classification of occupations and the INSEE occupational category (2003).**

| **Aggregate level (8 categories, 6 for people in the labor force)** | **Current publication level (24 categories including 19 for those in the labor force)** | **Detailed level (42 categories, 32 for people in the labor force)** |
| --- | --- | --- |
| 1 Farmers | 10 Farmers | 11 Farmers on a small farm  12 Farmers on a medium farm  13 Farmers on a large farm |
| Crafts workers, tradespeople, shopkeepers, and business heads | 21 Tradespeople and Crafts workers | 21 Tradespeople and Crafts workers |
|  | 22 Shopkeepers and equivalent | 22 Shopkeepers and equivalent |
|  | 23 Heads of businesses with 10 or more employees | 23 Heads of businesses with 10 or more employees |
| Managers and professionals | 31 Professionals and equivalent | 31 Professions |
|  | 32 Civil service managers, intellectual and artistic | 33 Civil service managers  34 Professors, Instructors, scientific occupations  35 Professions of information, arts, and entertainment |
|  | 36 Managers in companies | 37 Administrative and sales managers in companiesd  38 Engineers and production managers |
| 4 Intermediate occupations | 41 Intermediate white-collar occupations including teachers, health workers, civil servants, and equivalent | 42 School teachers, primary school teachers, and equivalent  43 Intermediate health care and social workers  44 Clergy, members of religious orders  45 Intermediate white-collar civil service office workers |
|  | 46 Intermediate white-collar office and sales workers in companies | 46 Intermediate white-collar office and sales workers in companies |
|  | 47 Technicians | 47 Technicians |
|  | 48 Foremen, supervisory employees | 48 Foremen, supervisory employees |
| 5 Lower-level white-collar workers | 51 Civil service office workers | 52 Civil service office and service workers  53 Police officers and military personnel |
|  | 54 Company administrative staff | 54 Company administrative staff |
|  | 55 Sales workers | 55 Sales workers |
|  | 56 Service workers providing direct services to individuals | 56 Service workers providing direct services to individuals |
| 6 Workers | 61 Skilled workers | 62 Skilled industrial workers  63 Skilled crafts workers  64 Chauffeurs  65 Skilled employees in handling, storage, and transportation |
|  | 66 Unskilled workers | 67 Unskilled industrial workers  68 Unskilled crafts workers |
|  | 69 Farm workers | 69 Farm workers |
| 7 Retirees | 71 Retired farmers | 71 Retired farmers |
|  | 72 Retired tradespeople, shopkeepers, and company heads | 72 Retired tradespeople, shopkeepers, and company heads |
|  | 73 Retired managers and intermediate white-collar occupations | 74 Retired managers  75 Retired intermediate white-collar occupations |
|  | 76 Retired lower-level white-collar and blue-collar workers | 77 Retired lower-level white-collar workers  78 Retired blue-collar workers |
| 8 Other persons not in the labor force | 81 Unemployed persons who have never worked | 81 Unemployed persons who have never worked |
|  | 82 Non-working diverse (other than retirees) | 83 Conscripts  84 Students and pupils  85 Diverse people not in the labor force and younger than 60 years (except retirees)  86 Diverse people not in the labor force and 60 years or older (except retirees) |
