# Supplementary material for: Does the availability of influenza vaccine at prenatal care visits and of immediate vaccination improve vaccination coverage of pregnant women?
Source: PLoS One. 2019 Aug 1;14(8):e0220705. doi: 10.1371/journal.pone.0220705 (PMC6675112; doi:10.1371/journal.pone.0220705)
Supplement: S1 Table — aOR: adjusted odds ratio; 95% CI%: 95% confidence interval. Ref.: reference. Adjusted for all sociodemographic variables and variables associated with perceptions of vaccines with p value <0.20 in univariate analysis. (DOCX) [file pone.0220705.s003.docx]

**S1 Table.**

**Sociodemographic determinants and pregnant women's perceptions of influenza and vaccination, multivariate analysis (N=248).**

| Variables | Vaccinated women  N = 48  n (%) | aOR | 95% CI |
| --- | --- | --- | --- |
| ***Maternity ward***  B and C, n=118  A, n=130 | 2 (1.7)  46 (35.4) | Ref.  22.89 | -  (4.89-143.93) |
| ***Geographic origin***  Other, n=134  Metropolitan France, n=114 | 14 (10.4)  34 (29.8) | Ref.  1.63 | (1.02-8.68) |
| ***Number of children***  None, n= 97  1, n=66  ≥ 2, n=85 | 27 (27.8)  10 (15.2)  11 (13.1) | Ref.  0.38  0.37 | -  (0.19-2.08)  (0.19-2.01) |
| ***Twin pregnancy***  No, n=239  Yes, n=9 | 44 (18.4)  4 (44.4) | Ref.  6.26 | -  (0.85-44.86) |
| ***Previous influenza vaccination****  No, n=195  Yes, n=51 | 27 (13.8)  21 (41.2) | Ref.  1.27 | -  (0.63-6.87) |
| ***Healthcare workers***  No, n=222  Yes, n=26 | 38 (17.1)  10 (38.5) | Ref.  0.93 | (0.29-5.41) |
| ***Social-occupational category***  Company heads, managers, professionals, n=66  Intermediate professions, tradespeople, crafts workers and shopkeepers, office, sales, and service workers, n=107  Farmers, blue-collar workers, unemployed, not in the labor force n=75 | 24 (36.4)  17 (15.9)    7 (9.3) | Ref.  0.35  0.35 | -  (0.19-1.86)  (0.13-1.98) |
| ***Consider they received enough information, n=70***  No, n= 178  Yes, n= 70 | 22 (12.3)  26 (37.1) | Ref.  1.93 | (1.11-10.35) |
| ***Sources of information***  None, n=44  In consultation, n=77  Family and friends, n=66  Media and internet, n=118 | 0 (0.0)  40 (51.9)  12 (18.2)  13 (11.0) | -  4.37  0.43  0.38 | -  (2.38-29.12)  (0.18-1.53)  (0.13-1.27) |
| ***Think that vaccines are***  Ineffective or not very effective, n=42  Effective or very effective, n=206 | 5 (11.9)  43 (20.9) | Ref.  1.24 | -  (0.32-6.03) |
| ***Think that the side effects of vaccines are***  Frequent or very frequent, n=93  Very rare or rare, n=155 | 9 (5.8)  39 (41.9) | Ref.  2.15 | -  (1.07-8.61) |

aOR: adjusted odds ratio; 95% CI%: 95% confidence interval.

Ref.: reference

Adjusted for all sociodemographic variables and variables associated with perceptions of vaccines with p value <0.20 in univariate analysis.
